# Nurse Specialist in the Organ and Tissue Donation Process with Coordination Role: A Scoping Review

**DOI:** 10.3390/nursrep15020039

**Published:** 2025-01-24

**Authors:** Donato Longo, Nicola Ramacciati, Gian Domenico Giusti

**Affiliations:** 1 Intensive Care Unit, “Vito Fazzi” Hospital, 73100 Lecce, Italy; donato.longo@asl.lecce.it; 2 Department of Pharmacy, Health and Nutritional Sciences, University of Calabria, 87036 Rende, Italy; 3 Medicine and Surgery Department, University of Perugia, 06100 Perugia, Italy; giandomenico.giusti@unipg.it

**Keywords:** transplant, nurse, specialist, donation, scoping review

## Abstract

**Background/Objectives**: In recent years, the introduction of specialist nurses, such as donation coordinator nurses, has been proposed as a potentially effective strategy to increase the number of donations and improve the quality of the process. However, experiences in this field are still limited. The aim of this study was to evaluate the impact of the inclusion of this professional in health systems, both in quantitative and qualitative terms. **Methods**: A scoping review was performed. Studies published from 1990 to 2024 were included regardless of the study design. The bibliographic search was performed on the databases MedLine, Web of Science, Scopus, CINAHL, and PsycInfo and on the search engines EBSCOhost, ProQuest, and Google Scholar. The search strings included keywords such as organ donation, transplant, procurement, and nursing role. The extraction and selection of articles were conducted in accordance with the PRISMA-ScR guidelines and with the methodology of the Joanna Briggs Institute for scoping reviews. The protocol study was prospectively registered with the Open Science Framework database on 3 December 2023, with registration number osf.io/wzxr3. **Results**: From the included studies, it emerged that the involvement of a nurse coordinator in the donation process is significantly associated with an increase in the number of organ donors and higher rates of family consent to donation. Additionally, the studies highlighted enhanced effectiveness in identifying potential donors and improvements in the training and preparedness of healthcare staff. **Conclusions**: The nurse coordinator in the donation process can have positive effects both on the management of the process and on the increase in the number of organ and tissue donations.

## 1. Introduction

To date, there is a great deal of interest and pressure on health systems worldwide to increase the number of organ and tissue donors [1] in response to the existing imbalance between demand and donated organs [2], especially due to the high prevalence of certain factors such as cigarette smoking, obesity, and alcohol consumption that contribute to the increase in chronic organ failure [3]. Therefore, since the early 2000s, the need for planning and programming by national governments and health services in this area has been highlighted [4,5].

The donation process is complex and requires the participation of multiple professionals [6,7] who must achieve a good level of collaboration in order to identify a potential donor in end-of-life care provided even outside of Intensive Care Units [8]. The identification of a potential donor is initiated following a death due to neurological (DBD) or cardiological (DCD) criteria [9]. All countries have strict protocol measures for proving BD and recognize that as mandatory notification to organ procurement institutions; furthermore, the majority of countries in the world have legislation for organ harvesting after cardiopulmonary arrest [10]. After the identification of a potential organ donor through these criteria, a medical evaluation is carried out to assess suitability for donation and define the patient as an eligible donor [11]. In order to convert an eligible donor into an actual donor, the success of the potential donor maintenance phase is fundamental. Donor optimization is an essential active process in organ donation that shifts attention from treatments focused on recovering the injured brain to those focused on treating potentially transplantable solid organs [12].

The European Union issued in 2008 an “Action plan for organ donation and transplantation” [13], which describes the priority actions with the aim of increasing the availability of organs, improving the efficiency and accessibility of transplant systems, the quality, and the safety, among which it includes the progressive appointment of coordinators for donations and transplants in all hospitals where there is a possibility of organ donation. The starting point could be the definition of a donation team composed of motivated and trained professionals [6]; in fact, the attitudes, experience, and competence of health workers are some of the main factors that influence the success of the donation process [14]. Training plays an important role, not only in improving technical skills, but also in increasing communication skills, particularly relevant in the field of donation [15], improving the outcomes in the decision of next of kin to donate [8].

The inclusion of a specialized nurse with a coordinating role in the organ and tissue donation process has been discussed for time. Some studies have focused on the inclusion of an institutionalized role of specialized, highly qualified, and trained professionals with responsibility for the results, and have highlighted the possibility that it can increase the performance levels in this area [16,17]. Nurses have, in their curriculum, the technical skills and organizational abilities to cover a coordinating role in the donation activity [18]. The role of Specialist Nurses in Organ Donation (SN-ODs) has been established as the leader of a multidisciplinary team [19], as in Spain, where they are called Nurse Transplant Coordinators (NTCs) [20], and in Great Britain, called Organ Donor Coordinator [18]; together with organizational and legislative strategies and a proactive strategy of the healthcare organization, positive results have been achieved in a short time in terms of an increase in donations [1,21] and improvement in the quality of donated organs [22], as well as a reduction in healthcare costs [23], especially for kidney transplants [6], and greater satisfaction for professionals [24].

The role of SN-ODs is highly specialized and requires technical, emotional, and organizational skills, and the ability to coordinate multidisciplinary teams and manage information and safety [20]. The SN-ODs seem to have a great impact on the donation process, especially on the decision of the deceased’s family to donate organs [1]. The key role of the nurse in coordinating the donation process is the same in the different geographical contexts and in the different names it takes on: it is focused on the patient and his/her family [25]; on the training staff of intensive care and emergency areas; in the early identification of a potential donor and in the implementation of the organ donation procedure; in the care of relationships with the bereaved family and with the institutions [1,16,23]; in promoting the importance of donation among colleagues, family, and community; in promoting clinical audits after a donation to reflect on the process and outcomes; in analyzing the performance data of their health company and comparing them with national data [26]; and, last but not least, in providing assistance in order to prevent and combat transplant tourism [5]. Nurses who fill this role have the same responsibilities and skills as other professionals who work in this area, such as doctors [18]. Though the figure of a specialized nurse in the organ and tissue donation process is not always represented by a nurse dedicated full-time to this activity and therefore also specifically remunerated, one donor per month would seem to be sufficient to cover the costs of 20 h per week for his work [23].

The general objective of this study is to identify outcomes in health organizations related to the inclusion of the specialized nurse in organ donation with a Donation Coordination role.

The secondary objective is to identify any gaps in knowledge that can support future research regarding a nursing specialization in this area.

## 2. Materials and Methods

This study is a scoping review. Scoping reviews aim to map what is present in the literature on a topic and define a body of knowledge, identify any gaps in research [27,28], provide a background [29], and understand the extent of knowledge present in an emerging field, including the heterogeneity of types of studies [30], without carrying out an in-depth analysis of the data obtained from each reference [31]. The starting point is the definition of the research question [32,33]. The recommended model for the construction of the research question for a scoping review is the PCC: Population, concept, and context [34]. For this study, the PCC was constituted as follows:

– Population: Organ and tissue donation from adult and pediatric patients.
– Concept: The presence of a specialized and dedicated nurse with a leadership role.
– Context: Both public and private health organizations.

This scoping review was conducted in accordance with the methodology developed by the Joanna Briggs Institute manual for knowledge synthesis [34] and the PRISMA-ScR guidelines [27]. An a priori protocol was defined, which was registered on the Open Science Framework database on 3 December 2023 with the reference osf.io/wzxr3 [35]. The protocol can be found via the following link: https://osf.io/e2wf5. The bibliographic search was performed on the databases MedLine, Web of Science, Scopus, CINAHL, and PsycInfo and on the search engines EBSCOhost, ProQuest, and Google Scholar. The MedLine database was consulted through the Pubmed search engine; the Web Of Science database was consulted through the Clarivate interface; and the Scopus database was queried on the Elsevier interface. The Mendeley Reference Manager software (®2.104.0-x64) was used to manage the studies obtained from the search. Studies published from 1990 to March 2024, from the published and unpublished literature, with any type of research, were included in the review. The search for evidence was limited to 1990 to have a good compromise between a comprehensive review and relevance to the health, social, economic, and cultural context in which health organizations work today. In fact, modern organizations for organ and tissue procurement were established between the late 1980s and the early 1990s [36,37]. The search strings included the following keywords: organ donor; donation; tissue; organ procurement; organ procurement; organ transplantation; organ harvesting; case manager; nurse specialist; nurse coordinator; and advanced nurse. The search was carried out in March 2024. The search queries used in the Medline database are as follows: (((((((organ donor) OR (donation)) OR (tissue and organ donor)) OR (tissue and organ procurement)) OR (organ procurement)) OR (tissue and organ transplantation)) OR (organ transplantation)) OR (tissue and organ harvesting) AND (((case manager) OR (nurse specialist)) OR (nurse coordinator)) OR (advanced nurse) AND ((improving) OR (efforting)) OR (increasing); publication date 1990 to 2024.

Studies that did not evaluate the role of the nurse coordinator in the donation process, studies focused only on tissue or blood donation, and studies published before 1990 were excluded. In accordance with the selection methodology reported by Livschitz and Dream [38], the reasons for excluding articles were reported at each step. The articles extracted were managed through the “Mendeley Reference Manager” software (®2.104.0-x64). Data extraction was completed by two researchers, with a review by a third researcher. Any discrepancies that occurred during the selection of studies were discussed between the researchers.

In this study, we adopted the methodology proposed by Kitchenham et al. [39] to evaluate the sensitivity and accuracy of our research. This approach, originally developed for the engineering field, defines these metrics by comparing the detected studies with a pre-defined set of known studies (gold standard). Sensitivity and accuracy are calculated to assess the comprehensiveness of the search strategy. Kitchenham et al. defined sensitiveness and accuracy as follows: sensitiveness = (KPF)/(KP); accuracy = (KPF)/(TPS); where KP corresponds to the number of known documents; KPF corresponds to the number of known articles found by the search process; and TPS corresponds to the total number of documents found by the search process. The gold standard that was defined for calculating the sensitivity and accuracy of research according to Kitchenham and colleagues was 42 studies: 19 articles found through a first non-standardized search and 23 articles contained in the bibliography of the article “The role of specialist nurses for organ donation” [1].

In accordance with the scoping review methodology [27,40], the assessment of the risk of bias and of the quality of the studies was not conducted. According to the Cochrane methodology [41], these data were collected from the studies: citation, first author, and date; country or region in which the study was carried out; date of data collection or study; analysis performed (if applicable); sample analyzed, no. (if applicable); mean age of the sample (if applicable); method of data collection; intervention performed; and outcome.

The outcomes considered in this scoping review were as follows: number of donors, identification of the potential donor, maintenance of the potential donor, interview with family members to obtain consent for donation, roles of the coordinating nurse, training, and economic aspects. The authors extracted data using the deductive method described by Pollock and colleagues [42]: the framework was decided during the definition of the protocol and the researchers extracted data according to this framework; the researchers read the articles, extracted the relevant information, and organized this information according to the established tables; and the researchers reviewed the data extraction ensuring that the information adequately described the framework. The data were collected independently by the authors after discussing the completeness of the tables.

## 3. Results

**Figure 1:**
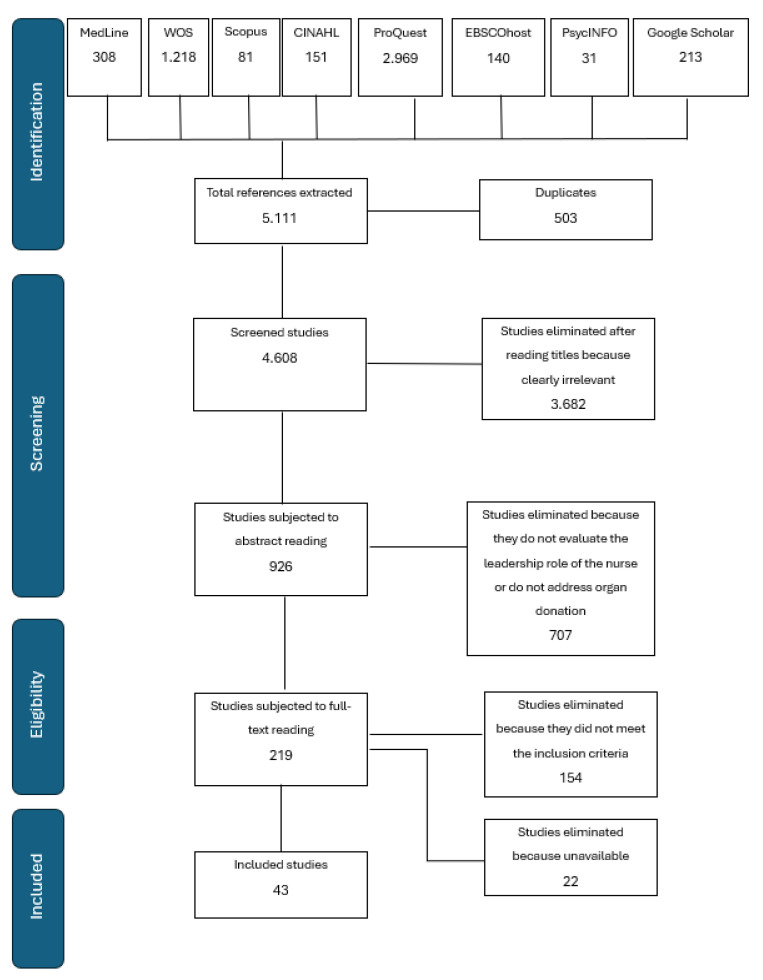
Study selection process.

The study selection is summarized in the following flowchart (Figure 1).

The study selection process is outlined in Figure 1. The exclusion of the 154 studies in the final screening had the following reasons: 103 studies were eliminated because they did not consider the nursing professional with a leadership role in the donation process, 33 studies were excluded because they did not evaluate the implementation in health companies of the nurse coordinator of the donation process, and 18 studies were discarded because they were not focused on organ and tissue donation.

At the end of the selection process, 43 documents were included in the scoping review. The articles included consist of 23 quantitative studies and 20 qualitative studies.

### 3.1. Evaluation of the Sensitiveness and Accuracy of the Search Process

The sensitiveness and accuracy of the search process were carried out according to the methodology of Kitchenham and colleagues [39] taking into account 42 references as the gold standard, of which 19 were obtained from a non-systematic search and 23 bibliographic references used in the study of Tocher and colleagues [1]. Of these 42 articles, 16 articles were found with this scoping review, showing a sensitivity of 38.0% and an accuracy of 37.2%:

$$Sensitiveness=\frac{16}{42}\times 100=38.0$$

$$Accuracy=\frac{16}{43}\times 100=37.2$$

where 16 is the number of gold standard studies retrieved and 42 is the number of studies contained in the gold standard, while 43 is the number of studies included in this scoping review.

If only the 19 articles extracted from the non-systematic search are considered (more specifically relevant for the topic of this scoping review), 14 out of 19 articles were found, with a sensitiveness of 73.7% and an accuracy of 32.6%:

$$Sensitiveness=\frac{14}{19}\times 100=73.7$$

$$Accuracy=\frac{14}{43}\times 100=32.6$$

where 14 is the number of studies of the second gold standard retrieved and 19 is the number of studies contained in the second gold standard, while 43 is the number of studies included in this scoping review.

### 3.2. Context-Related Aspects

**Figure 2:**
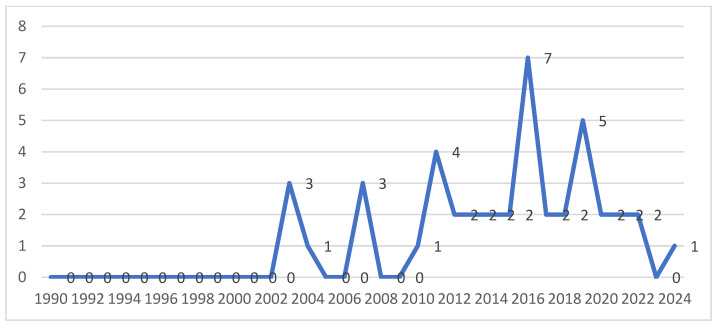
Distribution of studies by year of publication.

The studies included in the scoping review, as shown in Figure 2, were published in a time range from 2003 to 2023, with a substantial increase in publications starting from 2011; in fact, 81.4% of the selected studies were published from 2011 onwards.

Of the included studies, 12 (28%) were carried out in the United Kingdom; 8 (19%) in Brazil; 5 (12%) in the USA; 3 (7%) in France and Sweden; 2 (5%) in Italy, Poland, and Turkey; and 1 (2%) in Chile, Greece, India, Iran, New Zealand, and the Netherlands.

No data were collected regarding the effects of the presence of a nurse in the role of coordinating the donation process on the “maintenance” of the potential donor phase.

Among the studies included in the scoping review, 24 (56%) evaluated the effects of the presence of the coordinator nurse of donation process on the number of donors, 18 studies (42%) on the number of family members’ consents to donation, 14 (33%) on the reporting of the potential donor, 5 (12%) on the donation process, 11 studies (26%) evaluated the possible tasks that the coordinator nurse can perform, 2 studies (5%) evaluated the economic aspects related to the implementation of the process leader nurse, 2 studies (5%) evaluated the possible benefits of this professional on the staff training regarding donation, and 2 studies (5%) took into consideration the aspects related to the training of the donation coordinator nurse.

### 3.3. Number of Donors

A total of 24 studies evaluated the effect of the donation coordinator nurse on the number of donors [16,17,23,43,44,45,46,47,48,49,50,51,52,53,54,55,56,57,58,59,60,61,62,63]. Of these, 20 performed a pre-post-intervention evaluation of the implementation of the specialized nurse as an intra-hospital coordinator, all reporting an increase in the number of donors. Only three studies stated the opposite: Knihs and colleagues [49] highlighted that in Brazil, where the donation rate is low, the majority of the local coordinators are nurses, while in Spain, a leading country in the field of donation, the role is mainly covered by doctors [49]; two studies [51,52] reported that the donation rate was better in hospitals where the local coordinator was a doctor compared to those where the role was covered by a nurse.

Arsonneau [55] evaluated a procedure of early approach to family members by a specialized nurse in donation, reporting a 30% increase in the number of organs removed.

Mahdi and colleagues [63] evaluated the influence of the characteristics of the local coordinator (experience and profession) on the organ donation rate highlighting that the nursing profession can positively influence it; the same result was reported by Kumar and colleagues [61].

Overall, the studies reported an increase in the number of current donors from 26% to 113% after the implementation of the in-hospital nurse coordinator and an increase in the conversion rate from potential donor to actual donor from 22% to 77%.

### 3.4. Referring of the Potential Donor

The studies that evaluated the potential donor referring are 14 [17,23,43,44,46,47,48,50,51,52,54,55,58,64]. All of these reported an increase in the number of potential donor referrals after the implementation of the coordinator of the donation process nurse, with percentages ranging from 20% to 467.5%. Salim et al. [47] reported a 90% reduction in missed referrals after the institution of the in-hospital coordinator nurse; Ertin et al. [48] stated that if the person responsible for the donation process is a nurse, who is the professional closest to the patient, all cases of brain death could be referred, a concept also reiterated by Sikora et al. [52]. Arsonneau [55] reported that the procedure of an early approach to a donation by the specialized nurse can increase 20% in potential donor referring.

### 3.5. Consent of Next of Kin of Potential Donor

This issue has been focused in 18 studies [8,16,44,45,47,50,51,52,53,61,62,63,65,66,67,68,69,70]. Almost all of them (17 studies) reported an increase in the consent rate by family members following the implementation of the in-hospital coordinator nurse and a reduction in the number of family vetoes; only the study by Czerwiński and colleagues [51] reported an increase from 8.5% to 9.3% in the number of family refusals. In general, the consent rate by family members increased with a range from 8% to 37%. Gyllstrom et al. [45] highlighted that the request for donation made by the specialized nurse is not perceived as inappropriate by family members and, as reported by Hulme and colleagues [66], allows an increase in the consent rate. Two studies [67,68] evaluated consent to organ donation in the pediatric field and their results agree that the involvement of the specialized nurse in the family approach improves the consent rate for donation; Carone and colleagues [68] reported an increase in the consent rate from 66% to 85.7% when the child died within one week of admission.

### 3.6. Role of the Donation Coordinator Nurse

Harrison and colleagues [71] argue that every hospital that carries out donations should implement the role of a coordinator nurse.

Unlike Tocher and colleagues [1] who documented that the first transplant coordinator nurses were appointed in the UK in 1979, Harmanci and colleagues [64] reported that these appointments have been reduced in Turkey because the nurse can only hold, to date, the role of “assistant coordinator”. Aubrey [72] suggested a “two-tiered” approach, whereby the doctor treating the patient starts the discussion about a possible donation, which is continued by the specialist nurse after his/her role has been made explicit.

The areas of competencies of the donation process coordinator nurse identified are as follows: support to the relatives of the deceased in dealing with mourning and in the request for donation [1,48,55,73,74,75]; identification of potential donor [74,75,76]; support to staff members [74]; supervision, management, and coordination of the entire donation process [55,56]; responsibility for the care and maintenance of the potential donor [1]; collaboration in organ allocation [1]; recording of information about the potential donor and forwarding of documents and tests [76]; and daily visits or calls to departments where a potential donor may be present [75]. Ruta and colleagues [77] created a job description in which they defined the tasks of the donation nursing coordinator.

### 3.7. Donation Process

Five studies [56,61,70,78,79] focused on the possible advantages of the implementation of the donation coordinator nurse in the organ donation process. The coordinator nurse could bring effectiveness and humanity to the donation process [56] and speed up the process in order to reduce suffering and stress for the family members [70]. The specialized nurse in donation promotes the adequate planning of the entire process [61] and facilitates organization by reducing and controlling the times of the activities [79]. To achieve the results, it is necessary for the team to recognize the role of the nurse leader in the process [78].

### 3.8. Economic Aspects

Krekula and colleagues [53] reported advantages in cost-effectiveness, as the intra-hospital coordinator nurse allows for significantly increasing the number of donations. Silva and Silva and collaborators [23] documented an increase in hospital financial income of 40% and an investment return of 275%; the annual costs of investment were recovered in 4 months.

### 3.9. Training of the Donation Coordinator Nurse

Ruta and colleagues [77] defined the minimum requirements for access to this role in Italy, consisting of a degree in nursing and a training course related to donation and transplantation, communication, safety, and end of life. Tolfo and colleagues [80] highlighted in Brazil the lack of specific training and the use of self-taught training by nurses and exchanges of knowledge and experiences between novice and expert professionals.

### 3.10. Staff Training

Krekula and colleagues [53] reported a positive impact of the donation coordinator nurse on the training of ICU staff and on the introduction of new staff, as they can provide knowledge and support. Kumar and colleagues [61] highlighted the positive impact that the communication skills of the donation specialist nurse can have on staff attitudes towards this activity (Table 1).

## 4. Discussion

Organ donation should be an integral part and usual component of end-of-life care [81], in which nurses have an important role in reducing organ and tissue shortages [82] and through which they have the opportunity to save lives every day [83].

A scoping review was performed for this study, which is the research methodology suitable for identifying, mapping, or discussing some characteristics or concepts related to a topic [84]. The results of this scoping review highlighted eight themes related to the topic of interest: the effects of the presence of the donation coordinator nurse on the number of donors, the effects on the number of consents from the patient’s family members, the effects on the referrals of potential donors and on the whole donation process, the possible fields of action of the nurse leader in the donation process, the possible economic benefits deriving from the implementation of this professional, the effects on the attitudes and training of the staff with respect to donation, and the aspects related to the training of the donation coordinator nurse. The absence of studies that have focused on the maintenance phase of the potential donor can be explained by the fact that a nurse specialized in organ donation is the local coordinator of the donation process, while the care of the potential donor is generally carried out by a bedside nurse [85].

### 4.1. Evaluation of the Sensitiveness and Accuracy of the Research

The evaluation revealed a sensitivity of 38.0% and an accuracy of 37.2% when all the articles from the gold standard were considered. When focusing on the subset of articles from the non-systematic but topic-specific search, sensitivity increased to 73.7% while accuracy decreased to 32.6%. The lower sensitivity and accuracy may stem from the challenges in defining an optimal gold standard, a common issue in scoping reviews as highlighted by Pollock et al. [29]. Despite these limitations, the broader scope of scoping reviews, compared to systematic reviews, might explain these lower values.

### 4.2. Context-Related Aspects

The studies included in this scoping review were all published after the year 2000, highlighting the growth of interest in this new nursing competence over the last 25 years. On the other hand, organ donation and transplantation is a relatively recent activity and represents one of the extraordinary advances in medicine over the last sixty years, with particular growth since the 1990s with an increase in the effectiveness of immunosuppressive medicine. The observed increase in publications since 2011, with notable peaks in 2016 and 2018, aligns with significant advancements and heightened global focus on organ donation and transplantation during this period. A trend analysis of the leading transplantation journals from 2011 to 2021 indicates a 42.8% increase in the number of such journals, reflecting the field’s expansion and growing academic interest [86].

The role of a specialized nurse in donations and with coordination and leadership responsibilities has only gradually been increasing, and is still not implemented everywhere. This scoping review has documented the studies that have focused on this professional from the United Kingdom, Brazil, the United States, France, Sweden, Italy, Poland, Turkey, Chile, Greece, India, Iran, New Zealand, and the Netherlands.

Donation process coordinators are the key professionals in hospital settings who lead the donation process [51] and are its guarantors [87] in order to maximize the donation rate [88]. Transplant coordinators first appeared in the United States in the 1970s and were typically nephrology technicians or nurses who already worked in transplant units [89], but the country that first valorized nursing in the field of organ donation was the United Kingdom, establishing the figure of “Specialist Nurse in Organ Donation” (SN-OD) in 1979 [1] and since 2005 also introducing the “Specialist Requester” nurse to optimize the donation request [90]. In Iran, the country with one of the best donation programs in the Middle East [91], the change began with the enactment of the Brain Death and Organ Transplantation Act of 2000 [92]. Sweden started their first project to introduce a donation specialist nurse (DOSS) to support the intensive care staff in 2001 [53]. In Greece, the Local Transplant Coordinator, with responsibilities mainly in the identification and maintenance of the potential donor, was legally consolidated in 2002 and can be a nurse already working in intensive care [46], while in France the donation coordinator nurse is explicitly mentioned in a law of 2003 [56]. In 2005, Brazil established the Intrahospital Commission for Organ and Tissue Donation for Transplantation (CIHDOTT) consisting of a team of professionals, including a nurse who can act as coordinator [70] with responsibility for managing the whole donation process [23]. New Zealand began to define this role in 2007 [74], as did Turkey [48], while in Poland, the Ministry of Health funded the introduction of a local coordinator in all hospitals where the identification of the potential donor, the determination of brain death, and the procurement of organs is possible from 2010 [51]. Indian laws provided for the local coordinator to all matters relating to the procurement or transplantation of organs or tissues only starting from 2011, adding an amendment to the Transplantation of Human Organs and Tissues Act of 1994 [93]. In the United States, organ procurement and assistance to professionals and donor families is carried out externally to hospitals by 56 Organ Procurement Organizations (OPOs) coordinated by the Organ Procurement and Transplantation Network (OPTN) [94]. One of fifty-six OPOs was founded in 1989 from the collaboration of four local transplant centers and nurse Susan Gunderson, the first employee, which is granted full membership in UNOS as an organ procurement organization [95].

Spain has always been a leading country in organ donation [3]. In this scoping review, among the articles included, there are no studies focused on the Spanish context; this could be explained by the fact that although following a 2012 law the local coordinator can be represented by any adequately trained professional [96], this role in most cases is still covered by medical professionals [6].

Italy has long adopted many elements of the Spanish model [97]. To date, according to Italian legislation, the function of the local coordinator is carried out by a doctor; the local coordinator can avail of collaborators chosen from among the health professionals.

As can be seen from the results reported in Table 2, the role of the nurse coordinator is substantially comparable in the different geographical contexts and nurses find themselves facing similar daily challenges. Avril Wilson, a donor liaison sister at the Ulster Hospital said the following: “I work full time. Half my week is devoted to my sister’s duties in the unit, the other half to my role as donor liason sister through UK Transplant. We are using the Spanish system as a model. They have a donor liason person in each ICU” [73]. Evaldt et al. [70] reported the experiences of nurse coordinators of the donation process in Brazil, highlighting communication with the family members of the potential donor: “We cannot talk to the family, but trying to accompany that family, trying to understand the whole context that is inserted…, we already create a bond, a relationship with that family, welcoming, improving the reception, so that when the time comes, if necessary, there is already a bond of trust and we can talk better about the subject”. Evaldt et al. [70] also reported: “We support the medical team that will open the brain death protocol. We have the competence to certify that this brain death protocol is legal, through the documentation that is filled out during the clinical exams, apnea exam, and imaging”, and “We continue to accompany the patient to maintain this potential donor until the time of surgery”.

To correctly evaluate the differences between the methodologies for managing the donation and transplant system, it is appropriate to keep in mind the differences between the various health systems into which they are inserted [98]. An important component of national health systems is represented by the level of Universal Health Coverage (UHC), i.e., the possibility of access to quality services aimed at satisfying health needs, without incurring financial difficulties related to their payment. The World Health Organization (WHO) periodically defines the UHC Service Coverage Index on a scale of 1 to 100, which returned high values in 2021 for the countries from which the studies included in this scoping review come from [99], highlighting a possible correlation between the breadth of access to health services and the valorization of nursing. It is also important to consider the different methods used by countries with which people can declare their willingness to donate. In recent years, to improve the donation rate, some countries are moving to the opt-out system, which assumes the willingness to donate of all citizens in the absence of a declaration to the contrary, as Spain did in 1979 [100] and England did in 2020 [101]. Although the opt-out system is considered a promising strategy [102], in many countries a “soft approach” is used [103] continuing to consider the family’s consent as fundamental in the absence of declarations from the person assisted [104]. It should be noted that in countries where this scoping review has found a greater number of experiences of implementing the specialized nurse in donation, such as the United Kingdom and Brazil, the opt-in system is used, in which the ability to communicate with family members plays a very important role [105] and the nurse could have an advantage in the ability to establish an adequate relationship to obtain the family consent [48].

### 4.3. Number of Donors

Almost all the studies that analyzed the effects of the donation coordinator nurse on the donation rate (21 out of 24) [16,17,23,43,44,45,46,47,48,49,50,51,52,53,54,55,56,57,58,59,60,61,62,63] reported positive effects on the number of organ donors (eligible donors—a medically suitable person who has been declared dead based on neurologic criteria as stipulated by the law [7] and actual donors—a consented eligible donor [7]) resulting from the implementation of this professional in hospitals authorized for organ procurement. This quantitatively highlights that the donation process coordinator nurse could increase the number of donors.

The key to understanding these data could be traced back to the other themes identified by this scoping review, such as the nurse’s ability to obtain greater consensus from the family members, the ability to identify and refer potential donors, and the ability to optimally coordinate the donation process.

### 4.4. Referring of the Potential Donor

The studies reported an increase in referral rate after the implementation of the coordinator nurse of the donation process. The nurse could be the first professional to identify the potential donor thanks to the greater time spent with the patient compared to other professionals [106]. The possibility of identifying and reporting a potential donor is generally greater in the Intensive Care Units [107] and Emergency Departments [108], and in the future, pre-hospital care could also play a significant role, from which a significant number of referrals could arise [102]; in these settings, the nursing role is crucial and the quality of nursing performance is directly related to the realization of the donation [108].

### 4.5. Consent of Next of Kin of Potential Donor

A relevant factor in increasing the donation rate is the promotion of donation [82], an activity that the intra-hospital coordinator has to deal with with the patient’s family. On the topic of family consent to donation, 17 studies out of 18 [8,16,44,45,47,50,51,52,53,61,62,63,65,66,67,68,69,70] reported an increase in consent following the implementation of the donation coordinator nurse who carries out the request. Salim and colleagues [47] argued that the first way to increase the consent rate for donation is to improve the approach to the family; in this regard, it is appropriate to keep in mind that the nurse is the professional who establishes contact and a closer relationship with the patient’s family members [82] and has regular communication with both doctors and family members, so he could be an effective mediator between the parties, avoiding the fragmentation of communication and trying to find the best way to make the request [48]. da Silva Knihs and colleagues [109] identified three key aspects of communication with family members that the requester should not underestimate: the news of death, emotional support, and information on organ and tissue donation; it is, therefore, necessary for the requester to offer assistance that is also educational using appropriate language, integrating open conversations on the patient’s end-of-life wishes into daily conversations [110], and resolving family dilemmas [111], all aspects of the nursing profile.

### 4.6. Role of the Donation Coordinator Nurse

The presence of a coordinator nurse of organ donation could bring advantages by working in different phases of the process. Donation coordinator nurses play an important role in the donation process [105], in which they coordinate the staff [82], maintain contact with higher level centers, organize the process up to the organ procurement operations [72], refer the potential donor, maintain contact with laboratories for the necessary investigations and with the transplant team that is treating the recipient [57], coordinate clinical activities, and develop training courses for the staff and audits on the topic of donation [69]. It is important to underline that although the nurse coordinator of the donation process can take on different names (SN-OD [1], In-House Coordinator [50], transplant coordinator [51], Infirmier Coordinateur [55], or Nurse Transplant Coordinator [77]), this scoping review highlights that these figures perform the same functions and have the same skills in different contexts.

Although Aubrey suggests an early approach to requesting donation from family members [72], it should be kept in mind that the international literature agrees on starting the approach to requesting donation only after the family members have received the news of the patient’s death [7,10,91].

The donation process is naturally exposed to delays due to various factors, which could have negative effects on the donation, increasing the risk of deterioration of the donor’s organs or withdrawal of the donor’s organ, resulting in the loss of otherwise transplantable organs [103]; the nurse can help speed up the process as they have the organizational skills to bring efficiency, such as the ability to prevent the fragmentation of activities [112]. The studies included in this scoping review demonstrate that the nurse is perfectly qualified to carry out this role and, on the contrary, only the particular accreditation of the countries could alter the competencies of the nurse as a transplant coordinator, as reported by Harmanci and colleagues [64]. However, it is important that the nurse who holds this role has experience and training in the field of donation in order to improve the outcomes of the process [113].

### 4.7. Donation Process

This scoping review has highlighted that nurses specialized in donations could provide greater efficiency to the process thanks to the ability to plan and organize the different phases and activities that make it up. The coordination function has long been attributed to nursing in various settings, starting from the end of the 1970s [114], with levels of exclusive responsibility or linked to other responsible professionals [115], and represents one of the areas of advanced nursing expertise, together with clinical, research, training, and consultancy [116]. The coordinator plays an important role, as the donation process requires professional interdependence, cooperation, and integration between the members of the multidisciplinary team [108] and the nurse can be the professional able to plan the activities and manage the team, thanks also to the ability to create a bridge between the various professionals involved [117]. Building good relationships with the staff is an important aspect that the donation process coordinator must address [48] and this role can be carried out by the nurse since, as also stated by Fernández-Alonso and colleagues, the management of people and the multidisciplinary team is in itself an integral part of the nursing role [20]. Heeley and collaborators highlighted that the staff members view positively the figure of the specialized nurse in donation and consider it an important support in the implementation of the process [118].

### 4.8. Economic Aspects

Some studies highlighted the advantages deriving from the implementation of specialized nurses: Htay and Whitehead published a systematic review in 2021 in which they highlighted the advantages in cost-effectiveness of using specialized nurse professionals in healthcare companies [119], just as Fukuda and colleagues [120], who evaluated the possible advantages of specialized nurse in critical areas as coordinator in ICUs, highlighting the possibility of reducing costs related to assistance. As in other care processes related to transplantation [121], the outcomes of this scoping review reported advantages in cost/effectiveness deriving from the use of these figures with coordination in the organ donation process, highlighting a reduction in the costs of the process. This could be traced back to the nurse’s ability to identify and reduce complications early and to optimize procedures [122]. The cost-effectiveness and sustainability of healthcare companies in relation to the quality of care provided can bring advantages to companies and are, therefore, a crucial aspect of the development of nursing management figures [123].

### 4.9. Training of the Donation Coordinator Nurse

The results of this study highlighted a lack of structured training for local coordinators of the donation process. Many in-hospital coordinators have long expressed the need to receive more training. The heterogeneity of training quality for the in-hospital coordinator is one of the factors that influence the donation rate [124], which requires well-structured training courses [104], also including the acquisition of knowledge and experience from high donation rate centers [125] based in particular on the care of critical patients and the management of the whole process [126] and which allow the acquisition of skills in decision making, in creating effective relationships, and in managing conflicts and ethical dilemmas. It is important to consider that in the countries where organ donation is most successful, structured training courses have been implemented for healthcare professionals. Spain developed in 1991 the Transplant Procurement Management (TPM) program, which has become one of the largest and most international training programs [127]. In 2016, the European Union promoted a training course called EUDONORGAN, which included the “Train the trainer” program, dedicated to healthcare professionals, which was particularly successful from the point of view of nurses’ satisfaction [128].

### 4.10. Staff Training

Nurses, like other health professionals, are generally in favor of donation, even in the pediatric field, and state that discussion about it among staff members would be necessary every time a patient is approached at the end of life [129]; nevertheless, as also supported by Akbulut and colleagues [105], attitudes towards donation are strictly related to training and knowledge on the subject, and nurses express difficulties in the field of donation if they evaluate that their knowledge is not adequate despite having positive attitudes on this aspect of care [130]. Professionals, especially those with less experience, often have gaps in knowledge [131] which they find themselves having to fill independently [132]. The lack of knowledge regarding donation can reduce the quality of care and slow down or compromise the process [133] and can be the cause of the onset of burnout and compassion fatigue in staff, with increased turnover [134]. The specialized nurse in donation with a leadership role in the process could positively influence the training of the team, since, as also stated by Fowler and colleagues in a recent review, an expert manager influences the development of staff members and determines their performance [135].

### 4.11. Limits and Further Research

This study has some limitations.

One limitation of this study is the scoping review methodology itself. Scoping reviews, although useful for mapping knowledge on a topic in a broad way, do not include an in-depth critical assessment of the quality of the included studies. This means that the results obtained are not necessarily indicative of the robustness or reliability of the individual research analyzed; the absence of control groups in most studies included prevents isolating the results as a consequence of the intervention without uncontrolled variables. Another limitation is represented by the detection of sensitiveness and accuracy values of bibliographic search below optimal standards. Furthermore, a further limitation was found in the availability and accessibility of the selected literature; some articles were not available in full format, reducing the completeness of the analysis. This could have influenced the results, since potentially relevant studies may not have been considered in the synthesis. A further limitation of this study is given by the still-limited literature present on this topic. Furthermore, this nursing specialization is conditioned by the different international legal and professional contexts. The various geographical, organizational, and regulatory contexts, although providing a global overview, limit the possibility of drawing conclusions that can be applied homogeneously in specific healthcare contexts.

Although the results are promising, further studies can examine with greater scientific rigor the effectiveness of donation coordinator nurses, especially in contexts where the experiences of implementing the role of this professional are still limited. Furthermore, it would be desirable to conduct experimental studies that can isolate the factors that contribute to the success of donation and evaluate more precisely the impact of the coordinating nurse on different outcome indicators, such as the conversion rate from potential donor to actual donor and the quality of the organs removed.

## 5. Conclusions

This scoping review provided an overview of the importance of the coordinating nurse role in the organ and tissue donation process, highlighting how this figure can significantly contribute to increasing the number of donations, increasing family consent, and improving the attitudes and skills of the staff involved in the process.

The introduction of a specialized nurse with a leadership role can facilitate the management of the donation process, particularly in the phases of identifying the potential donor and communicating with family members. This study has highlighted that the nursing figure of the donation coordinator can speed up and make the entire process more effective by intervening in all its stages. Specific training of nurses is important, as it not only seems to increase their technical competence, but also improves their communication skills, a fundamental aspect for sensitively managing the delicate moment of the donation request. It is, therefore, important that the nurse leader of the donation process receives adequate training for the role they must cover.

The adoption of a structured organizational model, which fully integrates this figure into healthcare processes, could represent a significant step forward for the optimization of the donation and transplant system, with potential benefits for both public health and the economic sustainability of the healthcare system.

## Figures and Tables

**Table 1:** Summary of quantitative results.

Author-Year of Publication	Number of Donors	Actual Donors	Utilized Donors	Organs Retrieved	Referring of Potential Donor	Family Refusal
Arsonneau, 2016 [55]				+30%	+20%	
Czerwiński, 2014 [51]		+24% (from 868 to 1072)	+20% (from 2299 to 2754)	From 54% to 56%	+27% (from 1102 to 1400)	From 8.5% to 9.3%
da Silva Knihs, 2011 [49]	−74.6% compared to the Spanish model				+32.4% compared to the Spanish model	
Ertin, 2010 [48]	From 25 to 80				From 42 to 229	
Gardiner, 2017 [57]	+58% (from 809 to 1282)					
Garside, 2011 [17]			From 0 to 2 (*p* = 1.0)		From 3 to 26 (*p* < 0.0001)	
Gyllstrom, 2007 [45]		From 39% to 72% of potential donors				From 35% to 8%
Harmanci, 2011 [64]					From 60.2% to 47.5% after the new law with nurses only acting as assistants	
Hulme, 2016 [66]						74.4% of consent when nurse ask for donation
Karatzas, 2007 [46]		+132% (from 38 to 89)			+467.5% (from 40 to 227)	
Krekula, 2015 [53]		From 37% to 73% of potential donors				From 35% to 8%
Mahdi, 2024 [63]		+10.3%	+11.3%			39.37%
Meyer, 2019 [59]	No detailed data					
Mora, 2022 [62]		+32%				−29%
Noyes, 2019 [69]						From 54.2% to 39.0%
Oliva, 2019 [60]		From 31 to 51 (*p* < 0.002)				
Roth, 2003 [43]		From 15/year to 23/year, *p* = 0.0495)			+46% (from 84 to 124, *p* = 0.0495)	
Salim, 2007 [47]		From 34% to 50%		+17%	+39%	From 52% to 35%
Salim, 2011 [50]		From 63% to 77%			From 0.27/day to 0.35/day	From 18% to 6%
Sarlo, 2016 [54]		From 20% to 42%	+390% (from 26 to 128)		+132% (from 131 to 305)	
Scales, 2017 [67]						Variable rate across the centers, from 0% to 100%
Shafer, 2003 [16]	+31%		+48%			−28%
Shafer, 2004 [44]	+26%				+26%	−4%
Sikora, 2014 [52]	65% with doctor coordinatore vs. 25% with nurse coordinator	+113%			+100%	From 17% to 8%
Silva, 2015 [23]		statistically significant increase, no more data			statistically significant increase, no more data	
Silva, 2016 [58]		From 0.78/month to 1.60/month (*p* < 0.001)			From 3.05/month to 4.74/month (*p* < 0.05)	
Witjes, 2019 [8]						From 62.0% to 53.8% (*p* = 0.73)

**Table 2:** Summary of qualitative results.

Staff training	Staff education	The education of all staff in the unit on the organ donation process is important, to make them aware of what goes on and who can be a donor. (Bell, 2003) [73]
		Education and training of health care professionals is a key factor for success of organ donation. (Collins, 2012) [78]
	Staff support	The involvement of the multidisciplinary team, working in an integrated way, is important for the effectiveness of organ and tissue donation process. (Evaldt, 2022) [70]
		Nurses can play a key role in offering support and guidance to staff involved in organ donation. (Blythe, 2016) [74]
Consent of family members	Creation of a comfortable environment	During the family interview, the nurse provides a favorable and comfortable environment. (Evaldt, 2022) [70]
	Family members perception on specialized nurse	Family members who supported a donation decision identified the specialist nurse role as critical. (Noyes, 2019) [69]
		Family members appreciated the professionalism and integrity of the SNOD in the consent process. (Noyes, 2019) [69]
	Communication with family members	“It is to encourage people to explore their feelings and any doubts, but we would never, ever pressurise people”. (Bell, 2003, p. 2) [73]
		Nurses realize the importance of creation of a relationship of trust with the family members, associating this to a greater chance of obtaining the consent for donation. (Evaldt, 2022) [70]
	Request for organ donation	“The person closest to the family is the nurse and is the best person to ask the family if they know what their loved one feels about organ donation and if they have ever considered it consideration”. (Bell, 2003, p. 2) [73]
		The family interview was listed as an important competence of the nurse, since it can have a positive or negative outcome regarding the donation decision. (Evaldt, 2022) [70]
		Some SNODs found difficult to get the balance right between caring for the family and focussing on supporting the organ donation request. (Noyes, 2019) [69]
		The coordinating nurse has the contacts with the family and verify their understanding, consent or refusal to donate. (Arsonneau, 2016) [55]
		The involvement of the SN-OD in the approach to the family improves consent to donation. (Scales, 2017) [67]
	Attitude of Donation Coordinator Nurse	“The real success of the presumed consent legislation depend on specialist nurses carrying out their work under sensitive circumstances”. (Harrison, 2013) [71]
	Support to relatives	“My main responsibility is to make sure that, whenever appropriate, every potential donor family is given the opportunity to donate their loved one’s organs”. (Bell, 2003, p.1) [73]
		“A lot of support is given to relatives. In my position, I can be with relatives throughout. There’s a bond of trust and it’s such a privilege to be there with them”. (Bell, 2003, p.2) [73]
		Nurses provide the emotional support that is beneficial to families that are approached about organ donation. (Collins, 2012) [78]
		To increase the donation rate, welcoming family members is important needs; nurses recognize the importance of building a trusting relationship in increasing the possibilities of obtaining the consent for donation. (Evaldt, 2022) [70]
		Nurses offers important support and guidance to donor’s family members. (Blythe, 2016) [74]
Training of the nurse coordinator	Institutional training	“Updating health professionals is paramount, if we are to encourage and inform the public about organ donation”. (Blythe, 2016) [74]
		Nurses expressed a lack of training before take part of the commission and a gap in the professional training process. (Tolfo, 2018)
	Non-Institutional training	Responsible nurses must improve their qualifications through exchanges with more experienced personnel and field experiences. (Tolfo, 2018)
	Knowledge of Donation Coordinator Nurse	Nurses’ knowledge is essential for effective action in the process of organ donation and transplantation. (Tolfo, 2018) [80]
		knowledge enables the nurse to develop techniques, skills, create bonds and make ties between the elements that participate in the process of donation and transplantation. (Tolfo, 2018) [80]
Role of the coordinating nurse	Identification of potential donors	Nurses can play a key role in identification of the potential donor. (Blythe, 2016) [74]
		Nurses have a vital role to play in identifying and caring for potential donors. (Collins, 2012) [78]
		The nurse work in the active search for potential donors, which is essential to identify patients who may evolve to brain death and contribute to the organ donor process. (Evaldt, 2022) [70]
	Supervisory role	The coordinating nurse organizes and coordinates the donation and organ procurement activities. (Maroudy, 2016) [56]
		The coordinating nurse have a supervisory role of the organ donor process. (Arsonneau, 2016) [55]
	Organizational role	The duties of the donation coordinator nurse are: Planning of activities; Structuring and adoption of monitoring tools aimed at identifying and reporting of potential organ and tissue donors; Planning of standardized routes; Development of procedures, protocols and operating instructions; Procurement of organs and tissues; Communication of the data relating to the donor to the CRT for organ allocation; Compilation of the Local Register of Brain Injuries, of the samples taken, and of the causes of failure to make withdrawals; Promote and coordinate information, health education and growth activities cultural; Reporting, monitoring and classification of non-compliances, events and reactions adverse events detected in the donation and transplant process. (Ruta, 2021) [77]
		The coordinator nurse allows for adequate planning of the entire donation process, facilitating the organization of the transplant and reduces and controls the times of process activities. (Cailleton, 2016) [79]
		The functions of the SN-OD include ensuring the confirmation of death, approach the family to obtain consent for organ donation, support the family up to and beyond during the donation process, they are responsible for the care of the donor and the placement of the organs. It also collaborates with other transplant centers and coordinates communication with all members of the transplant team. (Tocher, 2019) [1]
	Bureaucratic and administrative role	The coordinator nurse is involved in bureaucratic and administrative activities. (Evaldt, 2022) [70]
	Care of potential donors	Nurses are responsible for the organization of care practice, in which they identify the needs, implement, evaluate, and monitor the results of the care provided to the potential donor. (Evaldt, 2022) [70]
Donation process	Process aspects	Nurses encourage speeding up the donation process, avoiding greater suffering and stress for family members. (Evaldt, 2022) [70]
		The coordinating nurse brings effectiveness and humanity to the process and allows the increase in the number of transplants. (Maroudy, 2016) [56]
	Organizational implementation of specialized nurse	In the emergency room, while waiting for the arrival of the SN-OD, the doctor treating the patient is the most suitable person to start the discussion regarding a possible donation; should therefore a “two-level” approach must be adopted. (Aubrey, 2013) [72]
		The role of the SN-OD must be clearly explained before the interview for the donation request. (Aubrey, 2013) [72]
		To improve the organ donation process it is essential that the role of SN-ODs is accepted as a vital member of the multidisciplinary team. (Collins, 2012) [78]
		Every hospital needs the implementation of the specialist nurse of organs donation. (Harrison, 2013) [71]
		Some nurses reported that there was no initiative of their own to join this role, but a personal appeal to assume the office; in other cases, professionals have banded together for the their interest. (Tolfo, 2018) [80]
		The presence of a nurse specialized in donation must to be essential for hospitals to be authorized to carry out organ donation activities. (Maroudy, 2016) [56]

## Data Availability

No new data were created or analyzed in this study. Data sharing is not applicable to this article.

## Public Involvement Statement

No public involvement in any aspect of this research.

## Guidelines and Standards Statement

This manuscript was drafted against the PRISMA extension for scoping reviews (PRISMA-ScR) [27].
